# Severe Complications After Endovascular Trapping of Vertebral Artery Dissecting Aneurysm: Simultaneous Occurrence of Medullary and Cervical Spinal Cord Infarction

**DOI:** 10.7759/cureus.21916

**Published:** 2022-02-04

**Authors:** Noriaki Matsubara

**Affiliations:** 1 Department of Neurosurgery and Neuroendovascular Therapy Center, Soseikai General Hospital, Kyoto, JPN; 2 Department of Neurosurgery, Tosei General Hospital, Seto, JPN

**Keywords:** internal trapping, endovascular intervention, spinal cord infarction, medullary infarction, vertebral artery dissecting aneurysm

## Abstract

Endovascular trapping of vertebral artery dissecting aneurysm (VADA) can lead to ischemic complications, including medullary infarction due to obstruction of perforating arteries, and cervical spinal cord infarction caused by ischemia of spinal arteries branching from the affected vertebral artery (VA). This report describes a rare case of concomitant medullary and spinal cord infarction following internal trapping of ruptured VADA.

A 47-year-old male presented with severe subarachnoid hemorrhage and neurological pulmonary edema, and a vertebral angiogram demonstrated VADA. A small-sized posterior inferior cerebellar artery (PICA) was found proximal to the affected vessel. The anterior spinal artery (ASA) branched distal to a dilated portion. Perforating arteries were insufficiently visualized due to image quality. Internal trapping was performed, and complete occlusion of VADA was achieved, preserving the origin of ASA. Postoperative magnetic resonance imaging (MRI) revealed ischemic lesions in the lateral medulla oblongata and upper cervical spinal cord. The patient presented with severe neurological symptoms, including lower cranial neuropathy due to medulla infarction, respiratory dysfunction, and tetraparesis due to cervical spinal cord infarction. The modified Rankin scale at three months was grade 5.

Various factors, including perforating artery ischemia, unstable general condition, and insufficient antithrombotic therapy, were considered the cause. Therefore, evaluating the tiny perforating and spinal arteries branching from the VA should be especially considered to avoid these complications. Furthermore, advances in angiographic apparatus and workstations should provide a high-resolution radiological image and adequate treatment strategy.

## Introduction

Intracranial vertebral artery dissecting aneurysms (VADAs) can be ruptured, which is associated with subarachnoid hemorrhage (SAH). They are followed by re-rupture, resulting in poor clinical outcomes [1,2]; therefore, early treatment is required to prevent re-rupture of the VADA. Therapeutic occlusion of the vertebral artery (VA) with endovascular trapping is a widely accepted treatment option for a ruptured VADA in SAH acute stage [2,3]. However, parent artery occlusion (PAO), such as internal trapping of VADA, can lead to medullary infarction due to obstruction of the perforating arteries branching from the affected VA [3,4]. Additionally, cervical spinal cord infarction is a rare but severe complication of internal trapping of VADA [3,5,6]. Additionally, spinal cord infarction is caused by ischemia of spinal arteries, including anterior and/or posterior spinal arteries. Therefore, in this report, the author describes a rare case of concomitant medullary and spinal cord infarction following internal trapping of ruptured VADA.

## Case presentation

A 47-year-old male healthy with no medical history presented with SAH; he collapsed during work and was transferred to our hospital by ambulance. He experienced cardiopulmonary arrest but was recovered by cardiopulmonary resuscitation. His level of consciousness upon arrival was E3V3M6 on Glasgow Coma Scale and II-20 on Japan Coma Scale. Additionally, he was slightly restless but followed some orders. His eyes had a right deviation, indicating impaired ocular motility, although he presented no apparent paresis in his extremities. His spontaneous breathing was maintained, and respiratory status was slightly irregular but preserved. Oxygen saturation of the peripheral artery (SpO_2_) was kept at a level of 90 using an oxygen mask. Therefore, he was not intubated at that time.

**Figure 1:**
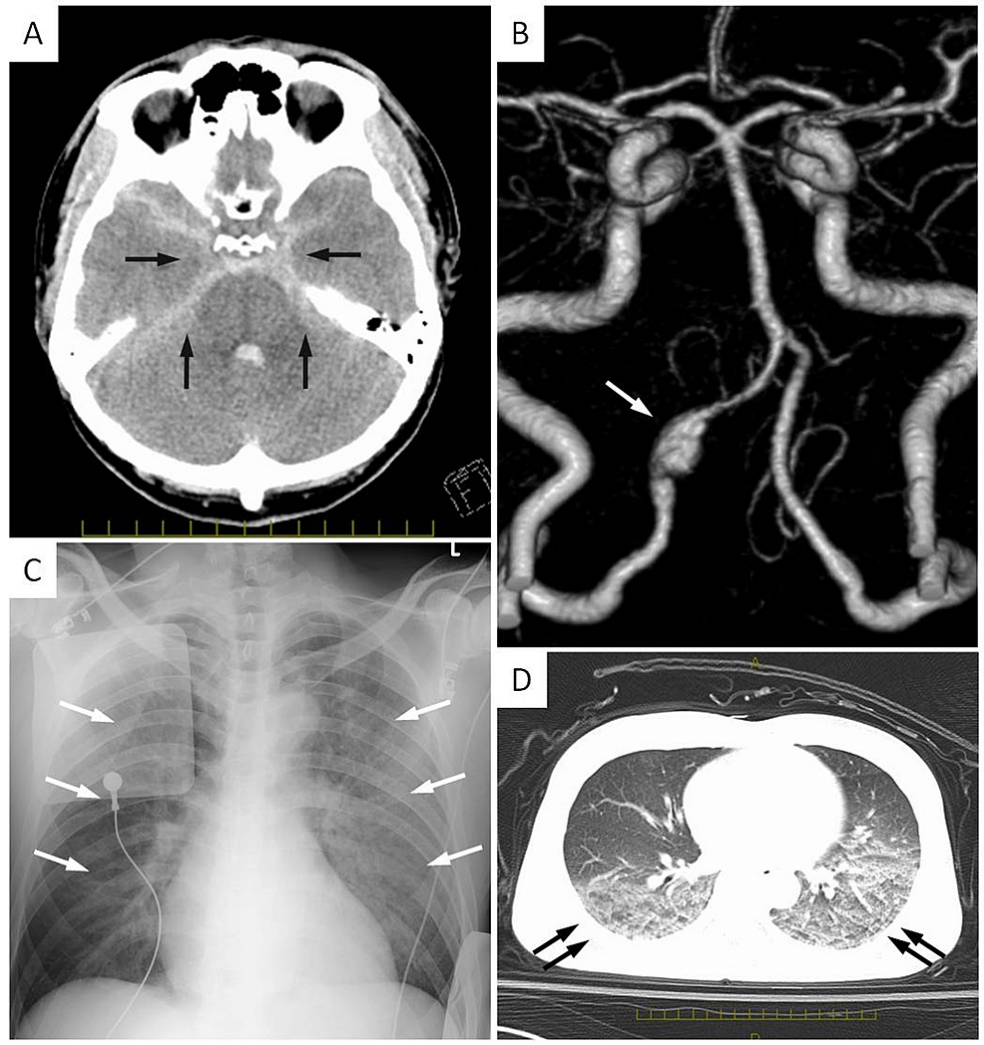
Initial radiographic images. (A) Head CT shows diffused subarachnoid hemorrhage in the basal cistern (arrows). (B) Three-dimensional CT angiogram shows the dilation of the right vertebral artery, indicating dissecting aneurysm (arrow). (C) Chest X-ray shows decreased permeability of the lung field (arrows). (D) Chest CT shows pulmonary infiltration in bilateral lung fields, indicating pulmonary edema (double arrows).

Head computed tomography (CT) revealed a thick SAH in the posterior cranial fossa. Chest X-ray showed decreased permeability of the lung field, and chest CT revealed pulmonary infiltration in bilateral lung fields, indicating pulmonary edema. CT angiogram (CTA) demonstrated dilation of the right VA, indicating VADA. The right posterior inferior cerebellar artery (PICA) was not described in CTA, and the left VA was equally sized (Figure 1). He was diagnosed with SAH of Hunt and Kosnik/WFNS grade IV, ruptured right VADA, and neurological pulmonary edema.

**Figure 2:**
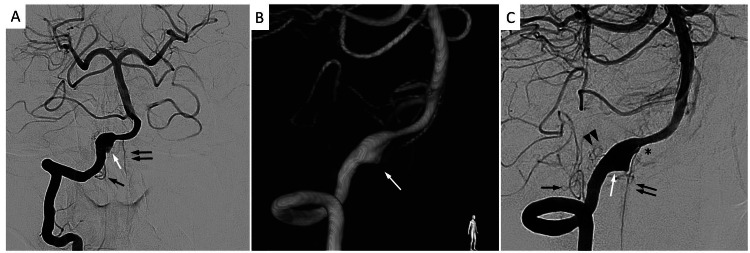
Preoperative angiographic images. (A) Right vertebral angiogram (anterior-posterior view) shows dissecting aneurysm (white arrow), ASA (double arrows), and hypoplastic PICA (arrow). (B) Three-dimensional angiogram (right oblique view) shows VADA (white arrow). ASA and perforating arteries are not well visualized in this image. (C) Right vertebral angiogram viewed from the same angle as panel B shows VADA (white arrow), ASA (double arrows), and hypoplastic PICA. ASA is distally located from VADA, and PICA is not involved with VADA. It is suspected that small perforating arteries are involved in the dissected segment in the vertebral artery but are unclear (double arrowheads). ASA, anterior spinal artery; PICA, posterior inferior cerebellar artery; VADA, vertebral artery dissecting aneurysm

On admission, an endovascular intervention was performed to treat ruptured VADA. An intervention was performed under local anesthesia and mild sedation with dexmedetomidine. After general heparinization, a sheath was inserted into the femoral artery, and a balloon guiding catheter was placed in the right VA. A conventional and rotational right vertebral angiogram was performed, and a right vertebral angiogram demonstrated VADA. Additionally, a small-sized PICA branching from the VA was found proximal to the affected vessel. The anterior spinal artery branched distal to the dilated portion of the VA. Perforating arteries were insufficiently visualized due to the image quality from the patient motion artifact. In a conventional angiogram, tiny arteries were shown around the dissecting segment but were unclear. Their directions and courses were difficult to evaluate despite a rotational angiogram (Figure 2).

Endovascular occlusion of VADA was selected for its curability, although possibility of ischemic event owing to perforating arteries ischemia was considered. Informed consent had been obtained from the patient’s family preoperatively.

**Figure 3:**
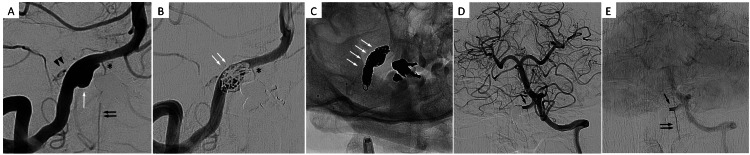
Endovascular internal trapping of vertebral artery dissecting aneurysm. (A and B) Right vertebral angiogram views of the working angle of endovascular internal trapping. ASA branches are distal to the VADA (white arrows). ASA origin is well visualized (asterisk). Framing coil masses (white arrows) were constructed, preserving ASA origin. (C) Plain X-ray obtained after intervention shows a mass of coils deployed in VADA and proximal vertebral artery (white arrows). (D and E) Left vertebral angiograms acquired after embolization. This shows retrograde filling of the right vertebral artery stump (arrow) and ASA flow (double arrow). ASA, anterior spinal artery; VADA, vertebral artery dissecting aneurysm

Following angiography, internal trapping of the VA, including dissecting lesion, was performed using detachable coils using the double catheter technique. Two microcatheters were navigated to the dissecting aneurysm. Also, under proximal flow control using balloon guiding catheter, framing coils were inserted via both microcatheters and were deployed, preserving the origin of ASA. Hereafter, the frame was filled with soft coils, and the affected vessel was filled back to proximally. A total of 18 coils were placed, and complete occlusion of VADA was achieved. Left vertebral angiogram showed blood flow to ASA branching from the right VA distal via VA union (Figure 3).

Furthermore, there were no procedural complications during the intervention, and the patient developed no additional neurological deficit after the intervention. Heparin was not reversed; a total of 3,000 units of heparin were intravenously administered during procedure). Postoperative anticoagulant/antiplatelet therapy was not given on the day of the intervention because of the risk of bleeding in a severe SAH case. Instead, ozagrel was administered from the day after the onset.

In a few hours postoperatively, his respiratory status gradually deteriorated, pink foamy sputum increased, and he could not sufficiently expectorate sputum. Therefore, his respiratory tract was congested with foamy sputum, and SpO2 was decreased to a level of 80 despite reservoir mask oxygen. He was intubated and treated using a ventilator under sedation for cardiorespiratory management. Unfortunately, his neurological symptoms could not be assessed afterward. The day after the intervention, the patient was sedated but he could communicate. He developed partial right hemiplegia and weakness in the left arm and leg, and his cough reflex disappeared.

**Figure 4:**
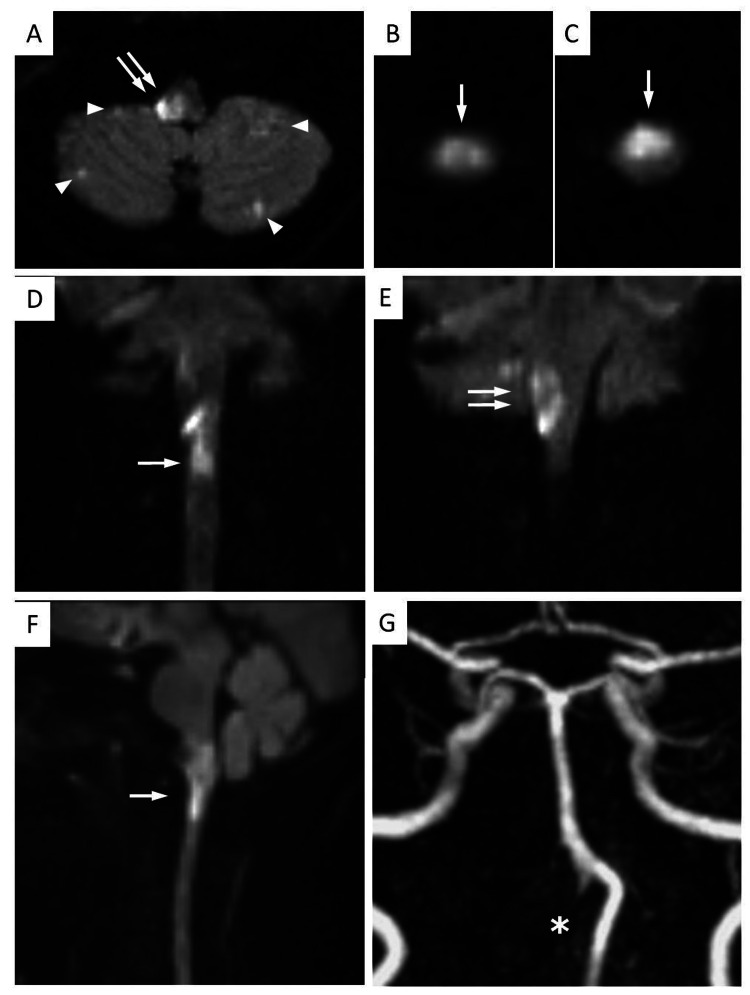
Postoperative MRI images. (A) Intracranial MRI (DWI) obtained three days after intervention shows right lateral medullary infarction (double arrow) and ischemic spots in bilateral cerebellum (arrowheads). (B and C) Cervical MRI (DWI, axial section) shows high intensities in the medial and anterior spinal cord (arrow). (D-F) Cervical MRI (DWI) (D, E: coronal, and F: sagittal view) shows right lateral medullary infarction (double arrow) and cervical spine infarction (arrow) at the level of C1/2. (G) MRA shows the right vertebral artery occlusion (asterisk).DWI, diffusion-weighted image; MRA, magnetic resonance angiography

Two days later, his respiratory condition improved, and MRI was performed. MRI diffusion-weighted imaging revealed high-intensity areas in the right lateral medulla oblongata and upper cervical spinal cord and multiple small high spots in the posterior circulation. The spinal lesion was medial-type ischemia, looking snake-eyed in several slices. While magnetic resonance angiography (MRA) showed dissected right VA occlusion, blood flow signal was also present in the distal stump of the right VA (Figure 4). MRI showed that his neurological symptoms were associated with the right medulla and spinal cord infarction.

After that, he presented with persistent severe neurological symptoms, including lower cranial neuropathy due to medulla infarction and respiratory dysfunction and tetraparesis due to cervical spinal cord infarction, although he was conscious.

He had presented with respiratory failure, and a tracheostomy was performed. Ventilator management was required for 2.5 months, and his tetraparesis persisted, although paralysis of left limbs improved considerably. Approximately three months after the onset, he was transferred to a rehabilitation hospital with a tracheostomy and gastrostomy. Notably, the modified Rankin scale at that time was grade 5.

## Discussion

The author presented this case to develop lateral medullary and cervical spinal cord infarction following internal trapping of ruptured VADA. To the best of my knowledge, this is the first case to simultaneously develop a rare complication with medullary and spinal infarction following endovascular intervention for VADA.

The prognosis of ruptured VADA is poor, and one reason is the high incidence of re-rupture from VADA [2]. Therefore, treatment for ruptured VADA in the acute stage of SAH is required. Also, endovascular intervention is the first-line therapy for ruptured VADA because of its less invasiveness [7]. Among the treatment options, PAO with internal trapping of the VA, including dissecting segment, is a feasible and durable treatment for VADA occlusion. Furthermore, deconstructive methods, including internal trapping, are curable with a low recurrence rate [3].

However, ischemic complications of PAO have been reported because of the occlusion of arteries branching from VA, such as perforating arteries and ASA. Most ischemic lesions are located in the medulla oblongata, and lateral medullary infarction is known as Wallenberg syndrome. Medullary infarction is a poor prognostic factor after internal coil trapping of a ruptured VA dissection [7-9]. Spinal cord infarction is a rare but severe ischemic complication after VADA trapping. Ischemia of ASA branching from the VA is the main cause of cervical spinal cord infarction. A few studies on posterior spinal artery ischemia have been reported, although it usually has good collateral networks and carries a small risk of infarction [3,5,6]. This case demonstrated lateral medullary infarction and medial spinal cord infarction.

Kashiwazaki et al. reported that spinal cord infarction occurs in 2.74% and perforating artery ischemia occurs in 9.59% of 73 ruptured/unruptured VADA [3]. Endo et al. reported that the incidence of medullary infarction is 28.9% in 90 patients (32% in 74 ruptured and 13% in 16 unruptured VADA) [4]. However, simultaneous medullary and spinal cord infarction events have not been demonstrated. Also, Tanoue et al. reported a radiological study of anatomical variations in perforating arteries from the VA. Non-PICA-type VAs give off more perforators than other types. This indicates that the trapping of non-PICA-type VAs is associated with a risk of ischemic complications [10]. In addition, Aihara et al. reported that proximal to PICA or non-PICA-type VADA presented a higher incidence of medullary infarction because proximal VA stump could not provide sufficient flow to small arteries branching from VA [8].

In this case study, PICA was hypoplastic and similar to non-PICA-type VADA, making the risk of medullary infarction high. Furthermore, even though ASA orifice was preserved in this case, spinal infarction occurred. The timing of the spinal cord infarction was not clear, but MRI obtained three days after intervention revealed it. This could result from hypoperfusion of ASA supplied from the VA stump. Due to neurogenic pulmonary edema, an unstable general condition might trigger ischemia in the brain stem and spinal cord. The initial poor clinical grade of SAH might influence the intracranial perfusion. Additionally, sparing use of postoperative antithrombotic therapy might worsen the ischemia of perforating arteries branching from VA and/or ASA originating from the VA stump. Therefore, perioperative antithrombotic treatment should be conducted to prevent delayed thrombosis of small vessels with decreased flow [7].

Internal trapping is performed to embolize the dissected segment of the VA, while avoiding the occlusion of PICA and ASA origins. Hence, the anatomical structure of VA, PICA, and ASA is assessed for interventional strategy. In contrast, it has been challenging to evaluate the morphological features of perforating arteries, although medullary ischemia has been considered to be caused by obstructing the orifice of unrecognized perforating arteries. These fine branches are not readily depicted on conventional angiography, such as in this case. However, recent advances in angiographic apparatus may provide high-resolution radiological and reconstructed images of three-dimensional rotational angiography to evaluate tiny perforating arteries [10,11]. In addition, adequate timing, volume, and injection rate of contrast medium in rotational angiography are important for obtaining precise images. Furthermore, general anesthesia can minimize motion artifacts, the most causal factor for unclear visualization. In this case, an intervention was performed under local anesthesia and sedation; therefore, the angiographic image provided here might have an insufficient resolution.

Reconstructive treatment, such as stent-assisted coiling, overlap stenting, or a flow-diverting stent, could be an alternative treatment method [12] if the origin of the perforating arteries is involved or close to the VADA during preoperative examination. However, these procedures carry a higher risk of recanalization or rebleeding than PAO. Thus, indications should be carefully considered, as preventing re-rupture is essential for VADA.

## Conclusions

This report presented a case with rare complications of lateral medullary and cervical spinal cord infarction after endovascular internal trapping of ruptured VADA. Various factors, including perforating artery ischemia, unstable general condition, and insufficient antithrombotic therapy, were considered as causes. Therefore, evaluating tiny perforating and spinal arteries branching from VA should be especially considered to avoid these complications. In addition, advances in angiographic apparatus and workstations should provide a high-resolution radiological image and adequate treatment strategy.
